# Unsupervised Learning Approach for Comparing Multiple Transposon Insertion Sequencing Studies

**DOI:** 10.1128/mSphere.00031-19

**Published:** 2019-02-20

**Authors:** Troy P. Hubbard, Jonathan D. D’Gama, Gabriel Billings, Brigid M. Davis, Matthew K. Waldor

**Affiliations:** a Department of Microbiology, Harvard Medical School, Boston, Massachusetts, USA; b Division of Infectious Diseases, Brigham & Women’s Hospital, Boston, Massachusetts, USA; c Howard Hughes Medical Institute, Boston, Massachusetts, USA; University of Michigan–Ann Arbor

**Keywords:** PCA, host-pathogen interactions, *in vivo* screen, principal-component analysis, Tn-seq, *Vibrio cholerae*, vibrio pathogenesis

## Abstract

Transposon insertion sequencing (TIS) is a widely used technique for conducting genome-scale forward genetic screens in bacteria. However, few methods enable comparison of TIS data across multiple replicates of a screen or across independent screens, including screens performed in different organisms. Here, we introduce a *post hoc* analytic framework, comparative TIS (CompTIS), which utilizes unsupervised learning to enable meta-analysis of multiple TIS data sets. CompTIS first implements screen-level principal-component analysis (PCA) and clustering to identify variation between the TIS screens. This initial screen-level analysis facilitates the selection of related screens for additional analyses, reveals the relatedness of complex environments based on growth phenotypes measured by TIS, and provides a useful quality control step. Subsequently, PCA is performed on genes to identify loci whose corresponding mutants lead to concordant/discordant phenotypes across all or in a subset of screens. We used CompTIS to analyze published intestinal colonization TIS data sets from two vibrio species. Gene-level analyses identified both pan-vibrio genes required for intestinal colonization and conserved genes that displayed species-specific requirements. CompTIS is applicable to virtually any combination of TIS screens and can be implemented without regard to either the number of screens or the methods used for upstream data analysis.

**IMPORTANCE** Forward genetic screens are powerful tools for functional genomics. The comparison of similar forward genetic screens performed in different organisms enables the identification of genes with similar or different phenotypes across organisms. Transposon insertion sequencing is a widely used method for conducting genome-scale forward genetic screens in bacteria, yet few bioinformatic approaches have been developed to compare the results of screen replicates and different screens conducted across species or strains. Here, we used principal-component analysis (PCA) and hierarchical clustering, two unsupervised learning approaches, to analyze the relatedness of multiple *in vivo* screens of pathogenic vibrios. This analytic framework reveals both shared pan-vibrio requirements for intestinal colonization and strain-specific dependencies. Our findings suggest that PCA-based analytics will be a straightforward widely applicable approach for comparing diverse transposon insertion sequencing screens.

## Abstract (precis)

Forward genetic screens are powerful tools for functional genomics. The comparison of similar forward genetic screens performed in different organisms enables the identification of genes with similar or different phenotypes across organisms. Transposon insertion sequencing is a widely used method for conducting genome-scale forward genetic screens in bacteria, yet few bioinformatic approaches have been developed to compare the results of screen replicates and different screens conducted across species or strains. Here, we used principal-component analysis (PCA) and hierarchical clustering, two unsupervised learning approaches, to analyze the relatedness of multiple *in vivo* screens of pathogenic vibrios. This analytic framework reveals both shared pan-vibrio requirements for intestinal colonization and strain-specific dependencies. Our findings suggest that PCA-based analytics will be a straightforward widely applicable approach for comparing diverse transposon insertion sequencing screens.

## INTRODUCTION

Transposon insertion sequencing (TIS) (variously referred to as IN-Seq, Tn-Seq, HITS, and TraDIS) (reviewed in references 1 and 2) is a powerful forward genetics tool for the identification of genetic loci contributing to bacterial growth in diverse environments (3–6). Since its introduction in 2009, the method has been applied to a wide variety of bacterial species which have been exposed to an even broader array of growth conditions, ranging from chemically defined media to the complex and poorly characterized milieu of host tissues during bacterial infection (7, 8).

TIS methodologies rely on generating “libraries” that contain large numbers of transposon (Tn) insertion mutants, followed by high-throughput sequencing to identify insertion sites, which enumerates the relative abundance of individual Tn mutants within the library. Three common applications of TIS are (i) essential locus analysis, in which genes with disproportionately low frequencies of Tn insertion are identified to make inferences of genetic essentiality (9, 10); (ii) genetic interaction studies, in which differential frequencies of Tn insertion libraries generated in different genetic backgrounds are used to infer suppressor and synthetic lethal relationships (2, 11, 12); and (iii) sequential selection studies (the primary focus of this work), in which changes in the relative abundances of mutants within a library before and after imposition of a selective pressure are used to infer the importance of each locus for growth under the selective condition (1, 2, 4, 8). Typically, the Tn libraries include multiple unique mutants for each locus. Although each mutant can be analyzed independently, often, data for all insertion sites within a locus are combined to mitigate site-specific effects.

To quantify a genetic locus’ contribution to growth in a sequential selection experiment, TIS studies usually calculate the fold change (between input and output libraries) in the relative abundances of insertion mutants mapping to that locus. Fold change between input and output libraries is calculated as (reads per gene in output library)/(reads per gene in input library). Loci with a log_2_(fold change) of <0 are considered depleted, and those with log_2_(fold change) of >0 are considered enriched. Several statistical approaches for analyses of TIS data have been proposed (2, 5, 13, 14), with different methods for normalization, modeling read count data, and combining data from multiple insertion sites within a gene.

In contrast to the variety of approaches for quantifying a locus’ contribution to growth in a single sequential selection experiment, there are few methodologies for comparing multiple TIS data sets. Jensen et al. (15) described a technique to compare various TIS data sets by first normalizing the fold change values against an experimentally derived population expansion factor prior to performing comparisons across screens. This approach allows a comparison of TIS data sets that can be adequately normalized; however, measurement of the population expansion factor can be challenging, particularly in *in vivo* experiments. DeJesus et al. (16) used a hierarchical Bayesian approach to incorporate the variation of the fold change value of each gene to identify genes displaying statistically significant differences between two TIS screens. They showed that such an approach permits the study of genetic interactions by comparing the results of screens conducted in parallel in two genetically different strains constructed from a single parental strain. While these two approaches provide valuable new tools, they have limited ability to compare the results of multiple TIS data sets, as both methods are restricted to pairwise comparisons.

Here, we present comparative TIS (CompTIS), a novel framework for conducting comparisons of multiple TIS data sets that relies on the dimensional reduction approach of principal-component analysis (PCA). As an unsupervised technique, PCA makes no prior assumptions about the structure of the data sets, providing an unbiased and broadly applicable approach to discovery. Dimensional reduction approaches such as PCA transform multivariate data sets into smaller sets of summary parameters while maintaining the underlying structure of the data sets, facilitating a direct interpretation of the relationships between data sets. Although extensively used in transcriptome sequencing (RNA-seq) and microbiome (i.e., 16S) analyses, PCA and other dimensional reduction approaches that extract the sources of variation between various multivariate data sets have not been thoroughly explored for comparisons of multiple TIS data sets. Given that the structure of TIS and RNA-seq data sets, which are comprised of matrices of genes and associated fold changes, are highly similar, we developed a PCA-based dimensional reduction approach for the comparison of TIS data sets.

CompTIS begins by implementing “screen-level” PCA and clustering to depict the variation between different screens and the relatedness of TIS data sets. This first step enables the grouping of screens with similar results without prior knowledge of experimental conditions, allows the identification of outlier screens, and facilitates the selection of comparable data sets for a subsequent “gene-level” implementation of PCA. Gene-level PCA examines variance across genes indicative of mutant growth phenotypes that are either consistent or divergent across TIS studies in order to identify genes that are important for growth under specific conditions or combinations of conditions. Here, we applied CompTIS to *in vitro* and several *in vivo* TIS data sets derived from studies of different pathogenic vibrio species and strains. This approach is not restricted to pairwise comparisons of TIS data sets and is not dependent on a specific upstream analysis method. CompTIS provides a general framework for unsupervised data discovery and meta-analyses of TIS studies.

## RESULTS AND DISCUSSION

### Background considerations.

Deriving biological insight from TIS data sets is complicated by their high dimensionality. Suppose we have *k* screens (in this work, our data sets contain up to almost a dozen screens, although larger ones are available [8]), each of which measures the fold change of *N* genes (typically in the thousands). For visualization purposes, we can represent each screen as one of *k* points in *N*-dimensional space, where the position along the *n*th axis is the log_2_(fold change) (L2FC) value of the *n*th gene. PCA identifies the line in the *N*-dimensional space along which there is the greatest variance among the *k* screens. Each screen is assigned a first principal component score (PC1), which is the position along this axis of greatest variance, and represents a weighted sum of the L2FC values for each gene. To compute the second principal component score, lines perpendicular to the line of greatest variance are identified (perpendicular so that variation in one principal component is independent from the others), and again, the line along which there is the greatest variance among the *k* screens is selected; the second principal component score for each screen is its position along this axis. The process is repeated, each time selecting the axis of maximum variance, subject to the constraint that it be perpendicular to all previous axes of maximum variance. Ideally, the variation in each screen can be accurately reconstructed by the first several principal components since they exhibit the greatest variance, and higher principal components can be dropped with little loss of accuracy.

We term the approach described above, assigning each of *k* screens as a point in *N*-dimensional gene space, a screen-level approach. Alternatively, we could assign each of *N* genes a point in *k*-dimensional screen space, which we term a gene-level approach. For the gene-level approach, we can also apply PCA, identifying the direction (in *k*-dimensional screen space) along which there is the greatest variation across genes and proceeding analogously. Whereas the screen-level approach facilitates the identification of patterns among screens or identification of screens of interest (outliers, for instance), the gene-level approach highlights patterns among genes and enables the selection of genes of interest.

### Screen-level PCA and clustering of TIS screens identify variation among replica screens and distinguish screens performed under different conditions.

We examined whether TIS data were amenable to PCA-based dimensional reduction and hierarchical agglomerative clustering by analyzing published data sets from sequential selection experiments. The data were derived from five screens performed with a high complexity Vibrio parahaemolyticus transposon insertion library (17). Of the five screens, four were biological replicates of screens carried out in infant rabbits, an animal model of intestinal colonization and diarrheal disease (18); the fifth screen was carried out *in vitro* in lysogeny broth (LB). These data sets were selected to test whether biological replicates of an *in vivo* screen have L2FC values for each gene more closely related to one another than to the values observed in an independent *in vitro* screen. The L2FC in the abundance of mutants corresponding to each locus was derived from output of the Con-ARTIST pipeline (13).

**FIG 1:**
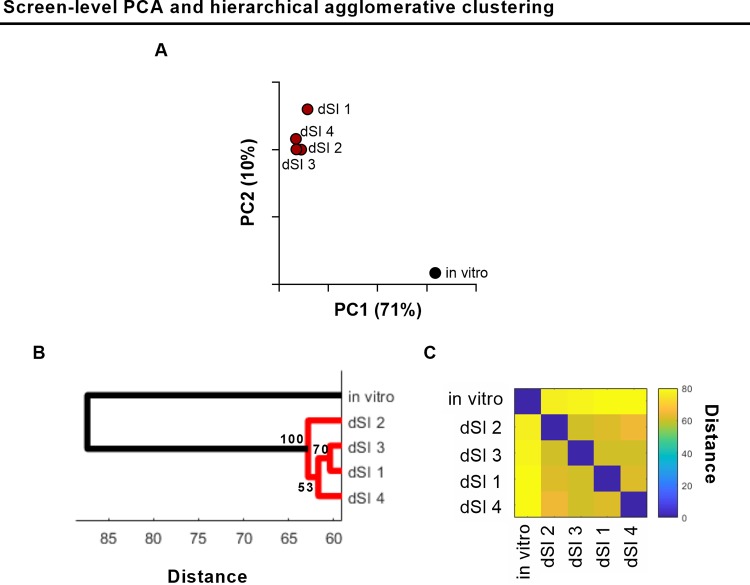
Screen-level comparative TIS analysis of V. parahaemolyticus screens. (A) Screen-level PCA of 5 V. parahaemolyticus TIS screens (1 *in vitro*, 4 *in vivo* biological replicates); units shown on axes are arbitrary values in principal component space. (B) Hierarchical agglomerative clustering with bootstrapping; values at each node represent approximately unbiased values calculated via pvclust; distal small intestine (dSI) 1 to 4 represent the 4 *in vivo* replicates. (C) Distance matrix of clustering.

We performed screen-level PCA to analyze the L2FC data for each variable (i.e., all of V. parahaemolyticus’ 4,830 nonoverlapping open reading frames) across all observations (i.e., 5 screens). For PCA, we employed a weighting scheme in order to minimize the impact of genes that, due to their relative lack of possible insertion sites, were intrinsically noisy (see Materials and Methods and Fig. S1 in the supplemental material). The first and second principal components accounted for 71% and 10%, respectively, of the overall variance across the 5 data sets. Thus, PC1 accounts for the majority of the variance in the data and clearly reveals the difference between the 4 *in vivo* experiments, which have similar PC1 values, and the *in vitro* experiment (Fig. 1A). PC2, while also separating screens by environment, also identifies variation among the *in vivo* screens.

10.1128/mSphere.00031-19.1 FIG S1 Weighting scheme for log_2_(fold change) values. A power law function (blue line) was fitted to the data (*r*^2^ = 0.96) as it best explained the relationships between the mean number of unique insertions per locus and the standard deviation of the L2FC values. This enables us to account for differential confidence in L2FC measurements for genes with different numbers of unique insertions. Download FIG S1, TIF file, 0.07 MB. Copyright © 2019 Hubbard et al. 2019 Hubbard et al. This content is distributed under the terms of the Creative Commons Attribution 4.0 International license.

The relatedness of the screens was also assessed via hierarchical agglomerative clustering of L2FC values, which provided additional support for the PCA-based groupings. We used a bootstrapping approach to determine the statistical support for the separation of the *in vivo* and *in vitro* data sets (Fig. 1B and C). Both approaches demonstrate that the variance between the *in vitro* observation and the four *in vivo* observations exceeds the variance between each of the 4 *in vivo* observations. Thus, PCA and clustering analyses of gene fold change values from multiple TIS data sets can reveal the relatedness of multiple sequential selection screens. These analyses could also potentially identify batch artifacts in the replicates, such as technical variation introduced during library preparation or sequencing, as they would appear as outliers. Both PCA- and clustering-based analyses of screens have merit. Clustering provides a bootstrap value to evaluate the robustness of each cluster, while PCA provides a more intuitive visualization of the relatedness of screens and/or replicates, particularly when analyzing a large number of screens.

### Gene-level PCA allows for integration of data across biological replicates of a screen.

Screen-level PCA enabled the visualization of the relatedness of TIS data sets and screens; however, it does not provide information about relationships among genes. To identify sets of genes whose mutants exhibit similar patterns across different screens, we implemented gene-level PCA of L2FC values from the V. parahaemolyticus data sets described above. The screen-level PCA (Fig. 1A) indicated strong divergence between *in vivo* and *in vitro* data sets, which we expected to dominate any comparison of these data, as conventional PCA is not robust for outliers. For this reason, and to provide a simple starting example, we restricted our analysis to the 4 *in vivo* screens. Since the screens are biological replicates, we did not expect to find complex patterns in the data but used them to illustrate the approach.

**FIG 2:**
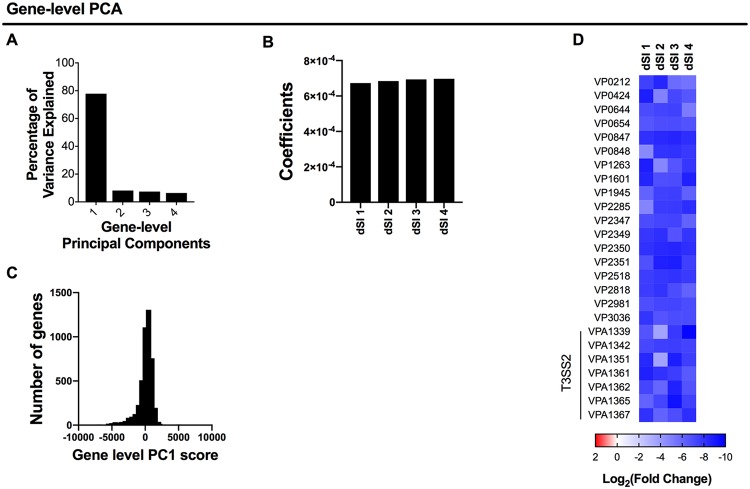
Gene-level comparative TIS analysis of V. parahaemolyticus screens. (A) Variance explained by each principal component in gene-level PCA of V. parahaemolyticus genes across 4 *in vivo* screens (distal small intestine [dSI]). (B) Principal component 1 coefficients. (C) PC1 score distribution across all genes. (D) Heatmap of log_2_(fold change) values for each *in vivo* replicate for the genes with the lowest 0.5% of gene-level PC1 scores. Genes in the T3SS2 gene cluster, a critical colonization factor, are highlighted.

Gene-level PCA provides principal-component scores for each gene, which are weighted sums of the L2FC measurements across the biological replicates analyzed. Each principal component has an associated set of weighting coefficients, which determine the contribution of each sample to the overall score per gene. The first principal component identified by gene-level PCA (PC1) accounted for 78% of the overall variance, while principal components 2 to 4 appeared to each account for a similar small amount of the remaining variance (Fig. 2A). Thus, our data were approximately one dimensional and hence well captured by a single quantity, PC1, for each gene. For PC1, the coefficients for each replicate had the same sign and were of similar magnitudes (Fig. 2B), so that the L2FC measurements from each sample contributed similarly to each gene’s PC1 score; that is, PC1 is a weighted average. The roughly equal weights of each screen are consistent with our expectation that gene-level L2FC measurements will be relatively consistent across biological replicates. Thus, PCA facilitates the comparison of replicates of screens by using weights informed by the data.

Most of V. parahaemolyticus’ 4,830 genes have PC1 scores close to zero, i.e., their corresponding transposon mutants did not exhibit a fitness defect *in vivo*, while a subset have very low scores (Fig. 2C). We generated a heatmap of L2FC measurements for genes comprising the lowest 0.5% of PC1 scores to further analyze the significance of these extreme values (Fig. 2D). These genes exhibited negative L2FC measurements across all 4 data sets and included several of the genes within the type III secretion system 2 (T3SS2) gene cluster, V. parahaemolyticus’ critical colonization/virulence factor (17, 18). Their low PC1 scores reflect the consistency and/or severity of mutant growth defects across biologic replicates of the *in vivo* screen. Collectively, these data indicate that PC1, which integrates biological replicates of an *in vivo* transposon screen, can provide a useful metric for the identification of genes required for intestinal colonization.

### Screen-level PCA reveals the relatedness of *in vivo* screens from different vibrio strains.

To determine whether CompTIS could be applied to more distantly related data sets, we repeated the screen-level and gene-level analyses described above after incorporating data from 7 additional *in vivo* screens (13, 19). Three of these screens utilized a transposon library constructed in Vibrio cholerae strain C6706 (a 1991 Peruvian isolate) (20), while the other four employed a transposon library constructed in V. cholerae strain H1 (a 2010 Haitian outbreak isolate) (21). The *in vivo* V. cholerae screens were carried out in an infant rabbit model (22), and the procedures used for infection and Con-ARTIST analysis were similar to those used in the V. parahaemolyticus *in vivo* screen. Importantly, our comparative TIS analysis was limited to the 2,356 protein-coding genes conserved across all three vibrio isolates (see Fig. S2A and B, Table S1). As expected, cluster of orthologous group (COG) analysis of this core gene set revealed that genes involved in translation/ribosome function and various metabolic and nutrient acquisition systems, such as transport of coenzymes, carbohydrates, nucleotides, and amino acids, were highly represented (Fig. S2C, Table S2). Thus, across these species and strains, genes involved in many metabolic functions have remained largely conserved.

10.1128/mSphere.00031-19.2 FIG S2 Core vibrio genes. (A and B) Prevalence of core vibrio genes present in V. parahaemolyticus, V. cholerae Peru and Haiti strains, and unique genes in V. parahaemolyticus and V. cholerae. (C) COG analysis of the core vibrio genes compared to the entire V. parahaemolyticus genome. Download FIG S2, TIF file, 0.7 MB. Copyright © 2019 Hubbard et al. 2019 Hubbard et al. This content is distributed under the terms of the Creative Commons Attribution 4.0 International license.

10.1128/mSphere.00031-19.5 TABLE S1 Core vibrio gene set between *V. cholerae* C6706, *V. cholerae* H1, and *V. parahaemolyticus* RIMD 2210633. Download Table S1, XLSX file, 0.10 MB. Copyright © 2019 Hubbard et al. 2019 Hubbard et al. This content is distributed under the terms of the Creative Commons Attribution 4.0 International license.

10.1128/mSphere.00031-19.6 TABLE S2 COG classification of core vibrio gene set and *V. parahaemolyticus* genome. Download Table S2, XLSX file, 0.3 MB. Copyright © 2019 Hubbard et al. 2019 Hubbard et al. This content is distributed under the terms of the Creative Commons Attribution 4.0 International license.

We wondered whether the screen-level PCA would be able to discern two anticipated results. First, that biological replicates of the same library exhibit more similarity in mutant growth phenotypes than those of distinct bacterial strains; and second, that the two V. cholerae data sets would more closely resemble one another than the V. parahaemolyticus data set, since C6706 and H1, related strains of the same species, are only distantly related to V. parahaemolyticus.

**FIG 3:**
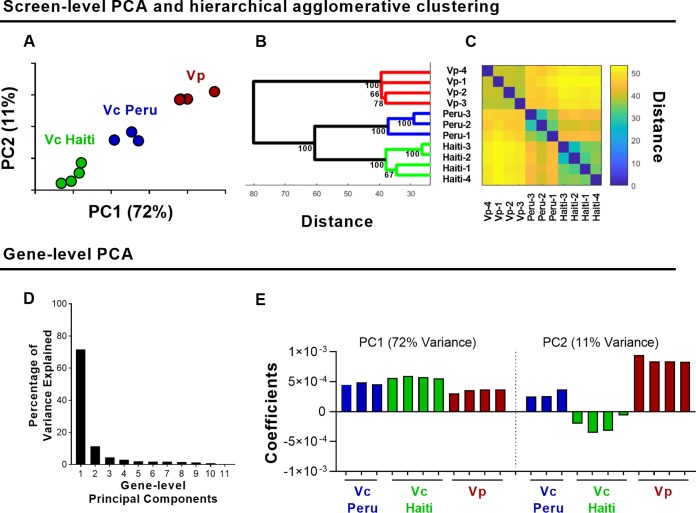
PCA-based analyses of *in vivo* TIS data from 3 pathogenic vibrio strains. (A) Screen-level PCA of V. cholerae C6706 (Vc Peru), V. cholerae H1 (Vc Haiti), and V. parahaemolyticus (Vp) *in vivo* screens; units shown on axes are arbitrary values in principal component space. (B) Hierarchical agglomerative clustering with bootstrapping; values at each node represent approximately unbiased values calculated via pvclust. (C) Distance matrix of clustering. (D) Variance explained by each principal component of gene-level PCA of all conserved vibrio genes across 11 *in vivo* screens. (E) Principal component 1 and 2 coefficients.

To assess the relatedness of these 11 data sets, we performed screen-level PCA to analyze the L2FC measurements for each variable (that is, conserved gene) across all observations (11 screens). In screen-level PCA, the first and second principal components accounted for 72% and 11%, respectively, of the overall variance. PC1 and PC2 values separated the data into 3 groups based on both species (V. parahaemolyticus versus V. cholerae) and strain variation (Peruvian versus Haitian V. cholerae) (Fig. 3A). Thus, in an unsupervised fashion, screen-level PCA grouped the data sets by strain, highlighting the power of this approach to detect patterns in large data sets. The normalization and weighting incorporated into the screen-level PCA reduces the variability between data sets and enhances the tightness of clusters (see Fig. S3). The inclusion of these two parameters enables PCA to separate the screens by organism in both PC1 and PC2 (Fig. S3). We note that each data set from a given species/strain represents a replicate selection of the same library; hence, library generation and the associated stochasticity of transposon insertions could explain some of the groupings we observe via PCA. Hierarchical agglomerative clustering confirmed the groupings found by PCA (i.e., there were species- and strain-specific clusters) (Fig. 3B and C). Notably, the two V. cholerae strains clustered into separate groups, suggesting that there are differences in the requirements for colonization in the core vibrio genes between these Peruvian and Haitian V. cholerae isolates. These two strains are both El Tor O1 V. cholerae, isolated only 19 years apart from each other, a short time relative to the length of time that V. cholerae has been evolving with humans. Thus, the screen level PCA revealed unexpected differences in the genetic requirements for colonization of two closely related V. cholerae strains, even when restricted to core genes that are largely conserved. The effect of minor genetic variation between closely related strains can manifest in various ways, including differences in gene expression levels. Recently, Zhao et al. (23) reported that there are differences in the expression levels of several loci linked to intestinal colonization between these two strains, raising the possibility that their distinct requirements for colonization are explained by gene expression differences.

10.1128/mSphere.00031-19.3 FIG S3 Effect of normalization and weighting on screen-level PCA. Screen-level PCA run on the 11 vibrio screens with the following parameters: (A) no normalization of the data (norm) or weighting on the data (weights), (B) normalization of the screens only (no weighting), (C) weighting of the genes (no normalization of the screens), (D) normalization and weighting (also shown as Fig. 3A). Download FIG S3, TIF file, 0.5 MB. Copyright © 2019 Hubbard et al. 2019 Hubbard et al. This content is distributed under the terms of the Creative Commons Attribution 4.0 International license.

### Gene-level PCA identifies both strain-independent and strain-dependent mutant growth phenotypes.

Although V. parahaemolyticus and V. cholerae rely on independent virulence factors to access and proliferate within their respective niches within the small intestine, we sought to identify common *in vivo* genetic requirements for colonization across pathogenic vibrios by carrying out gene-level PCA on all 11 *in vivo* vibrio screens (see Table S3). The first and second gene-level principal components accounted for 72% and 11% of the overall variance, respectively (Fig. 3D). The PC1 coefficients for each data set were of similar signs and magnitudes (Fig. 3E), indicating that all strains contribute similarly to PC1 scores. That is, PC1 constituted a roughly equally weighted average of L2FC measurements across the 11 data sets, approximating the average L2FC value of a gene across the screens analyzed (see Fig. S4). Notably, the weighting, rather than being assumed to be equal or provided by an independently determined population expansion factor, is determined by the data itself. Thus, a majority of the overall variance across these screens is strain independent, suggesting that the core vibrio genes required for survival/growth in the small intestine are conserved across the two major pathogenic vibrios, V. parahaemolyticus and V. cholerae. Therefore, even though these pathogens depend on species-specific virulence factors for colonization, T3SS2 for V. parahaemolyticus and toxin-coregulated pilus (TCP) for V. cholerae (18, 22), they also share many genetic requirements for *in vivo* growth. In contrast, the PC2 coefficients were consistent across biological replicates but differed between screens by both signs and magnitudes, thereby reflecting strain-dependent phenotypes (Fig. 3E). Interestingly, PC2 reflected discordance between the Peruvian and Haitian V. cholerae strains, evident in the differences in signs of the coefficients. Further validation/dissection of the genes that account for these differences is warranted. In addition, these observations suggest that studies comparing the genetic requirements for host colonization among closely related strains can highlight unexpected strain-specific dependencies.

10.1128/mSphere.00031-19.4 FIG S4 Gene-level PC1 represents an average of the log_2_(fold change) values across the screens analyzed. Comparison of gene-level PC1 score per gene to the average log_2_(fold change) value of each gene, averaged across all 11 screens; linear regression plotted in dashed red line, *r*^2^ = 0.84. Download FIG S4, TIF file, 0.2 MB. Copyright © 2019 Hubbard et al. 2019 Hubbard et al. This content is distributed under the terms of the Creative Commons Attribution 4.0 International license.

10.1128/mSphere.00031-19.7 TABLE S3 Gene-level principal-component analysis scores for core vibrio genes analyzed. Download Table S3, XLSX file, 0.5 MB. Copyright © 2019 Hubbard et al. 2019 Hubbard et al. This content is distributed under the terms of the Creative Commons Attribution 4.0 International license.

**FIG 4:**
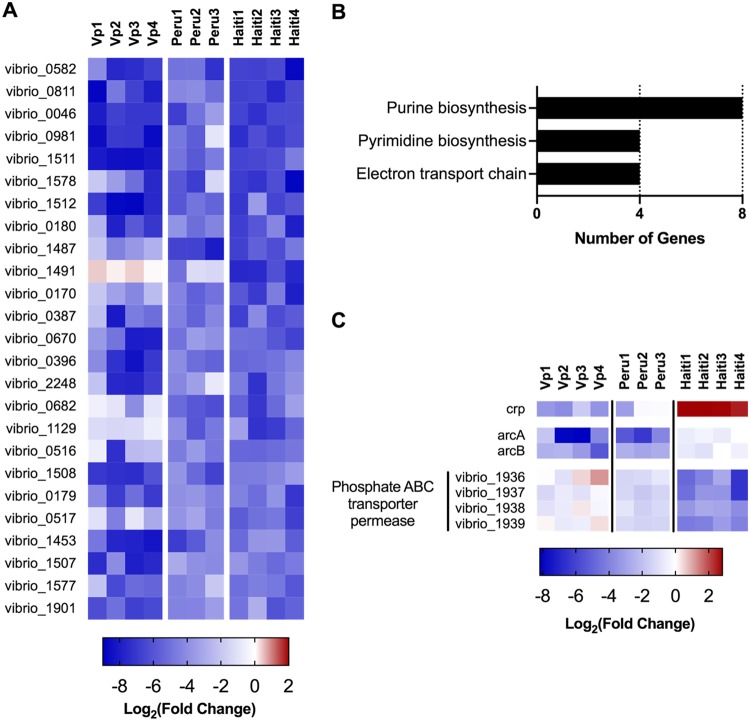
Gene-level PC1 and PC2 identify genes required for colonization by all strains and by specific strains, respectively. (A) Heatmap of log_2_(fold change) values of genes with the 1% lowest of gene-level PC1 scores across 11 *in vivo* vibrio screens. (B) Categories highly represented among the genes with the lowest 1% of PC1 scores (25 genes total). (C) Heatmap of a subset of genes with discordant L2FC values across strains, selected from genes with the lowest 1% or highest 1% of gene-level PC2 scores.

Heatmaps were generated to visualize the lowest 1% of PC1 scores (Fig. 4A). These genes generally exhibited negative L2FC measurements across all 11 data sets and thus display strain-independent attenuation *in vivo*. Functional analyses revealed that the majority of these genes are involved in *de novo* purine and pyrimidine nucleotide synthesis as well as complex 1 of the electron transport chain (Fig. 4B). These observations suggest that access to nucleotides in the small intestine is limited for both pathogens, even though they modify the host environment in distinct ways; e.g., V. parahaemolyticus, in contrast to V. cholerae, causes marked disruption of the intestinal epithelium (18). Thus, even though the niches that V. parahaemolyticus and V. cholerae occupy in the small intestine likely differ, both pathogens rely at least in part on shared genes to proliferate in the host environment. Collectively, these findings demonstrate that gene-level PCA can facilitate the identification of genes required for growth *in vivo* (or in other environments) by multiple bacterial strains. Furthermore, analyses of PC2 and potentially other principal components can yield information regarding species- or strain-specific growth requirements as well, such as the differential reliance between the Peruvian and Haitian V. cholerae strains on the cAMP receptor protein (Crp), the ArcAB two-component system, and an uncharacterized phosphate ABC transporter permease for growth *in vivo* (Fig. 4C). The mechanisms that underlie these unexpected strain-specific *in vivo* growth requirements warrant further investigation.

### Summary and conclusions.

We developed CompTIS, which utilizes screen-level and gene-level PCA and clustering, to accomplish meta-analysis of TIS data. Screen-level PCA distilled genome-wide mutant growth phenotypes to facilitate comparisons across screens. This unsupervised learning method was capable of establishing the relatedness of screens, distinguishing replicate screens from those conducted in different experimental contexts, and identifying outlier screens. Furthermore, clustering analysis with bootstrapping corroborated the PCA analysis and enabled the identification of statistically significant clusters. Using such an approach, we detected differences in the genetic requirements for intestinal colonization in two closely related strains of V. cholerae.

The second part of our approach relied on using gene-level PCA to identify variance across genes indicative of mutant growth phenotypes that are either consistent or divergent across multiple screens. Importantly, gene-level PCA does not depend on *a priori* hypotheses regarding consistency or divergence of mutant growth phenotypes across screens for the identification of significant gene sets. Instead, the utility of gene-level PCA lies in its capacity to guide the formation of hypotheses regarding the genes that modulate growth, both in biological replicates and in separate strains and environments.

In summary, our findings suggest that a PCA- and clustering-based analytic approach provides a straightforward method for comparing the results of different TIS screens, thereby facilitating the discovery of novel associations between screens and guiding hypothesis development for additional experimentation.

## MATERIALS AND METHODS

### Weighting of Con-ARTIST log_2_(fold change) measurements.

We used previously published TIS screens for our analyses (13, 17, 19). To minimize the influence of noise due to variability in log_2_(fold change) (L2FC) measurements observed across genes with few unique insertion mutants, the L2FC measurement for each gene was weighted based on the variability observed in genes with similar numbers of unique insertion mutants. This procedure ensured that low-variability (i.e., high-confidence) observations were given proportionally higher weights than those with higher variability (see Fig. S1 in the supplemental material).

For each screen, we calculated the standard deviation of each gene’s L2FC value by comparing the L2FC values calculated for each gene across the 100 independently simulated input libraries that are generated during the Con-ARTIST analysis (13). Note that for our data sets, for each gene, the average fold change is calculated by averaging the ratio, (reads per gene in output library)/(reads per gene in simulated input library), across all simulated input libraries. The input libraries are generated via multinomial-based resampling in order to model stochastic drift, i.e., a bottleneck, in the input library, hence limiting the effect of genetic drift on downstream analysis and reducing the number of false-positive findings (13). We fit a power law function (*y* = *ax^b^*) to the standard deviation of each gene’s L2FC value and the number of unique insertion mutants represented in each gene. Fitting was performed using the Fit function in Matlab (Curve Fitting Toolbox) with the following parameters: power1 and name-value pair Robust and Bisquare. We found that a function with *b* of ∼−2 fit the data well. For each screen, each gene’s weight was calculated by first using the generated coefficients to determine the estimated standard deviation in L2FC values based on the number of unique insertion mutants present for the gene and then taking the inverse of the estimated standard deviation (i.e., for gene q, its weight, w_q_ = 1/(*ax^b^*); where *x* is the number of unique insertion mutants present for the gene).

### Principal-component analysis.

Prior to performing PCA, we removed genes from the analysis that contained one or more uncalculated L2FC values (e.g., arising when there were no reads mapping to the gene in a particular screen). Next, the L2FC values in each screen were standardized (i.e., z-score normalized) using the zscore function in Matlab. In this final normalized L2FC matrix, which was used for PCA analyses, rows corresponded to genes and columns corresponded to screens.

**(i) Screen-level PCA.** Weighted PCA was performed in Matlab using the PCA function with the default algorithm (single value decomposition [svd]), “centered” set to off, “VariableWeights” corresponding to a column vector of the sum of the calculated weights of each gene across the screens being analyzed, and “Weights” corresponding to a row vector of the sum of the calculated weights of all the genes in each screen. Screen-level PCA was performed on the transpose of the normalized L2FC matrix.

**(ii) Gene-level PCA.** Weighted PCA was performed in Matlab using the PCA function with the default algorithm (single value decomposition [svd]), “centered” set to off, “VariableWeights” corresponding to a row vector of the sum of the calculated weights of all the genes in each screen, and “Weights” corresponding to a column vector of the sum of the calculated weights of each gene across the screens being analyzed. Gene-level PCA was performed directly on the normalized L2FC matrix.

### Clustering and bootstrapping analysis.

We used the normalized L2FC matrix to perform hierarchical agglomerative clustering with bootstrapping using the pvclust package (version 2.0-0) (24, 25) in R (version 3.3.2) (26) and the following parameters: distance function, Euclidean; clustering method, Ward's (ward.D2); and *n* = 1,000 bootstrap replications. pvclust provides two *P* values, the standard bootstrap probability and the adjusted unbiased (AU) value, which is calculated using multiscale bootstrap resampling and represents a more unbiased *P* value than the bootstrap probability.

### Identification of conserved vibrio genes.

The V. cholerae N16961 genome (which differs from the C6706 Peru strain by only several single nucleotide polymorphisms [SNPs]) was used to identify unique V. cholerae H1 homologs (using the V. cholerae KW3 genome) of N16961 genes that exhibited >90% nucleotide identify across >90% of gene length. A subsequent protein blast of these conserved V. cholerae genes against the V. parahaemolyticus RIMD 2210633 genome identified unique V. parahaemolyticus homologs that exhibited >50% amino acid identity across >80% of gene length. Ultimately, 2,356 genes met these standards of conservation across the three vibrio strains.

### Cluster of orthologous groups analysis.

COG analysis of the V. parahaemolyticus RIMD 2210633 genome was performed using the functional annotation (COG) function of WebMGA (27). To facilitate comparison, percentages of genes in each COG category were calculated for the whole genome (4,830), and for the subset of V. parahaemolyticus genes that consisted of the core vibrio gene set (2,356). Figures and heatmaps were made using Matlab and GraphPad Prism 8.

### Data availability.

Matlab scripts for running the screen-level and gene-level PCA analyses can be accessed at https://bitbucket.org/gabriel_billings/comptis.
