# Supplementary figures and images for: Unsupervised Learning Approach for Comparing Multiple Transposon Insertion Sequencing Studies
Source: mSphere. 2019 Feb 20;4(1):e00031-19. doi: 10.1128/mSphere.00031-19 (PMC6382967; doi:10.1128/mSphere.00031-19)

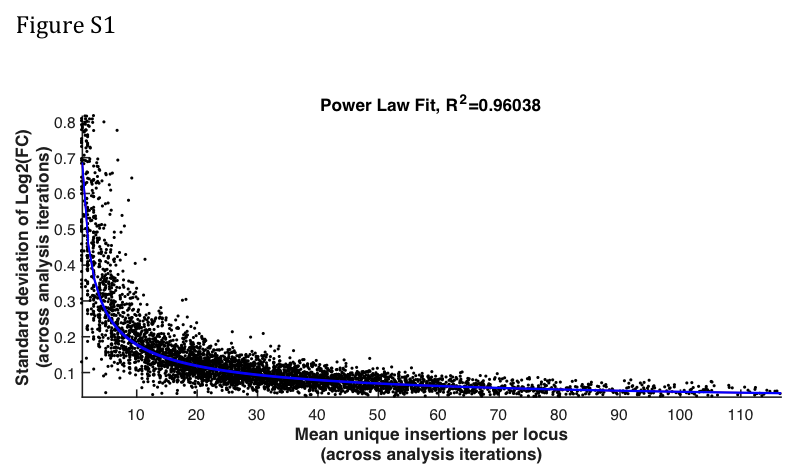

Supplement: FIG S1 [file mSphere.00031-19-sf001.tif]

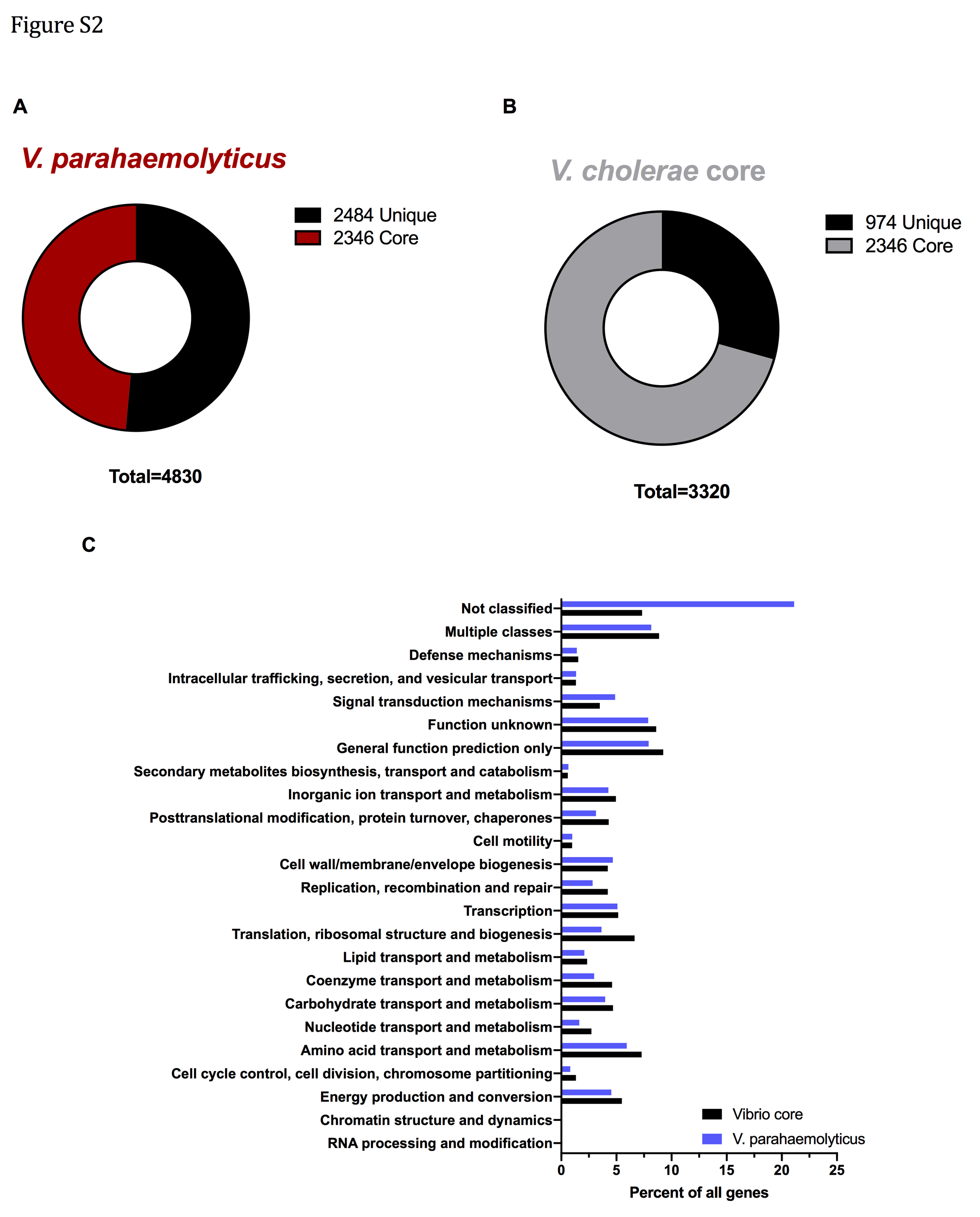

Supplement: FIG S2 [file mSphere.00031-19-sf002.tif]

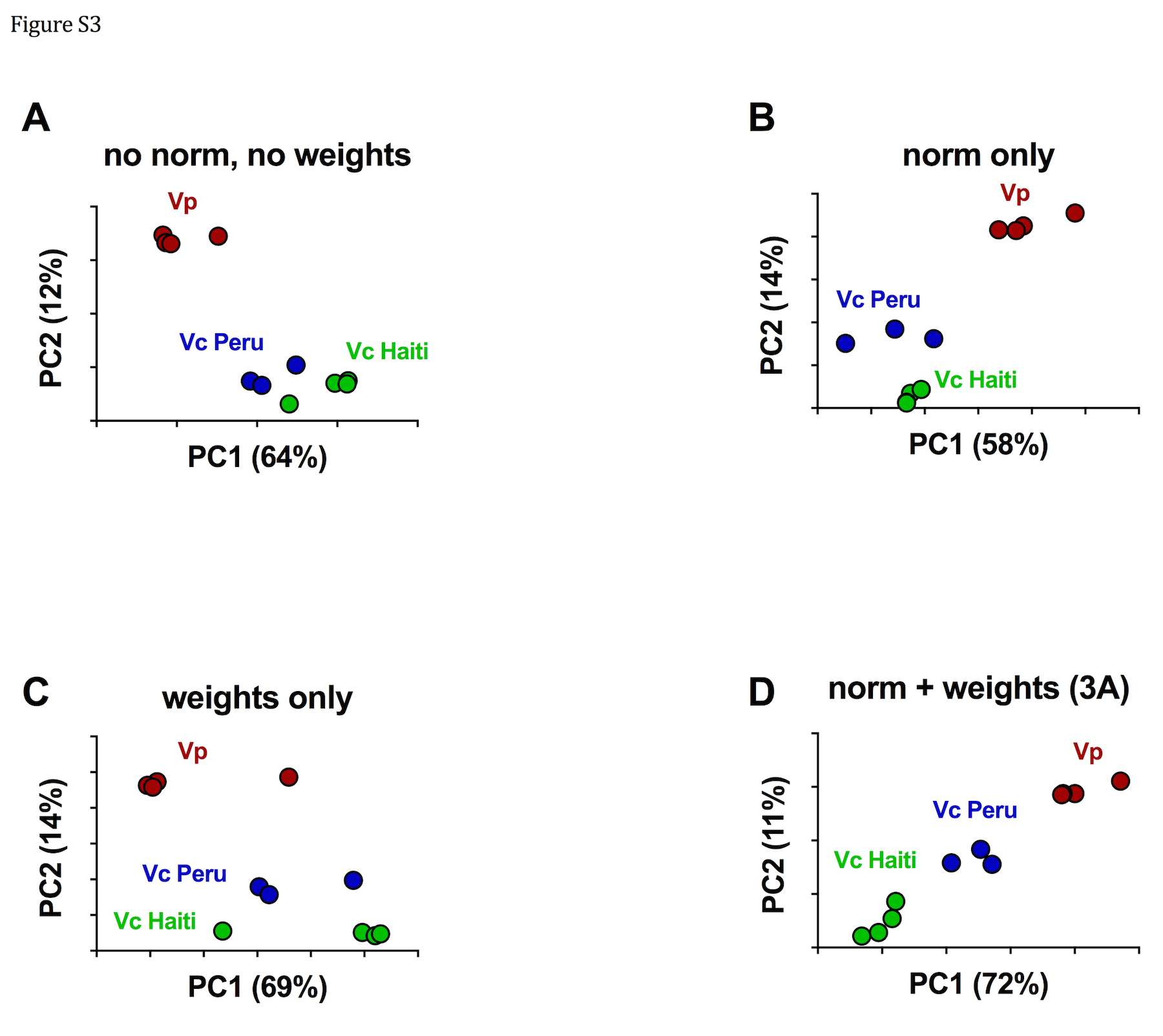

Supplement: FIG S3 [file mSphere.00031-19-sf003.tif]

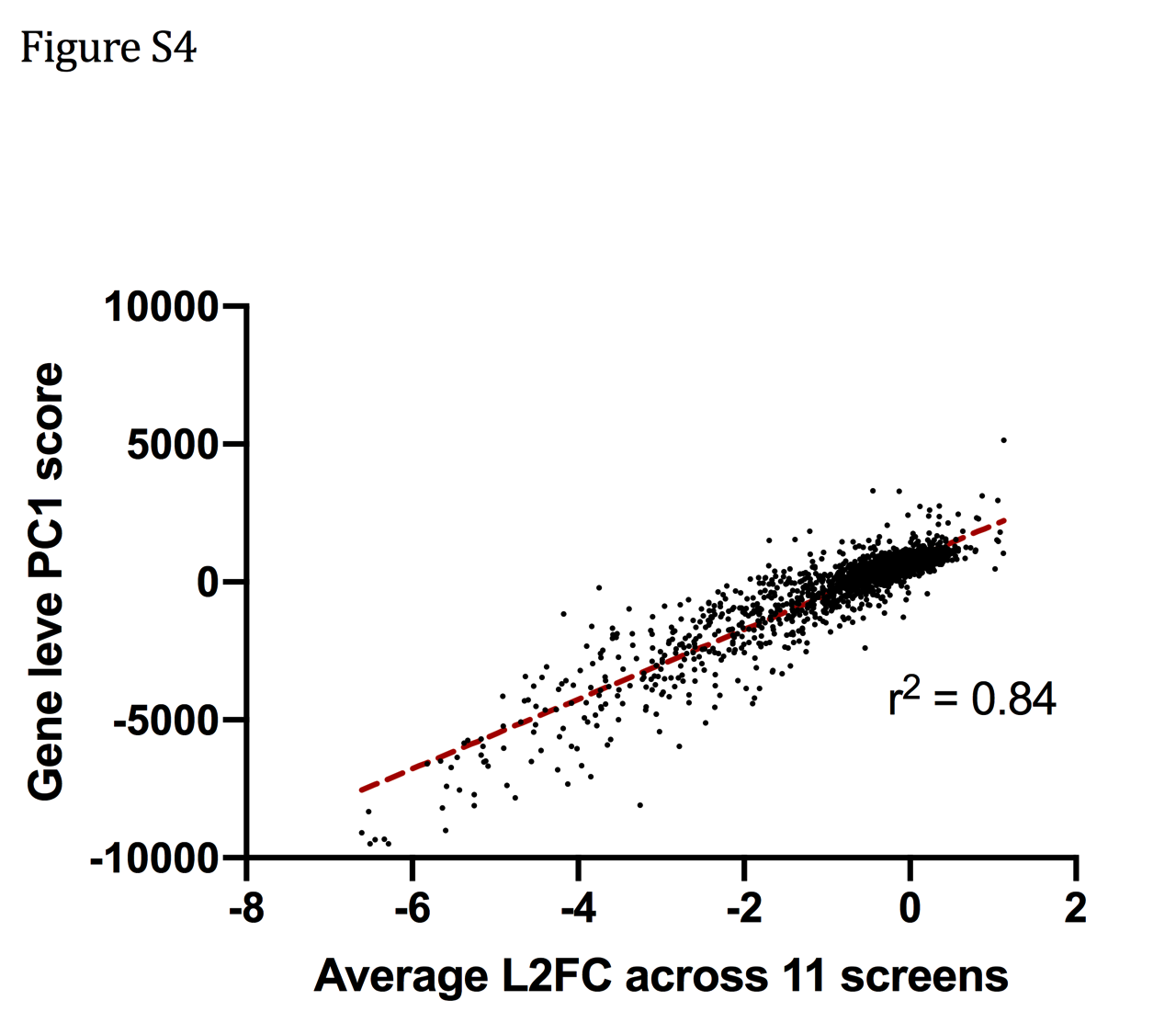

Supplement: FIG S4 [file mSphere.00031-19-sf004.tif]
